# The *SLC6A3* gene polymorphism is related to the development of attentional functions but not to ADHD

**DOI:** 10.1038/s41598-020-63296-x

**Published:** 2020-04-10

**Authors:** Katarzyna Kuc, Maksymilian Bielecki, Ewa Racicka-Pawlukiewicz, Michał B. Czerwinski, Anita Cybulska-Klosowicz

**Affiliations:** 1 Department of Psychology, SWPS University of Social Sciences and Humanities, Warsaw, Poland; 2 Department of Child Psychiatry, Medical University of Warsaw, Warsaw, Poland; 3 Laboratory of Neuroinformatics, Nencki Institute of Experimental Biology, Polish Academy of Sciences, Warsaw, Poland; 4 Laboratory of Neuroplasticity, Nencki Institute of Experimental Biology, Polish Academy of Sciences, Warsaw, Poland

**Keywords:** ADHD, Attention

## Abstract

Neuropharmacological and human clinical studies have suggested that the brain dopaminergic system is substantively involved in normal and pathological phenotypes of attention. Dopamine transporter gene (*SLC6A3*) was proposed as a candidate gene for Attention-Deficit/Hyperactivity Disorder (ADHD). We investigated the effect of the *SLC6A3* variants on cognitive performance in ADHD and healthy children and teenagers. Participants completed cognitive tasks measuring attentional switching, selective and sustained attention, and effectiveness of alerting, orienting and executive attention. We estimated the effects of 40 bp variable number of tandem repeat (VNTR) polymorphism located in the 3′ untranslated region (3′ UTR) (9-repeat vs 10-repeat allele) of the *SLC6A3* gene, ADHD diagnosis, age, and their interactions as predictors of cognitive performance. ADHD children demonstrated deficits in most of the examined attention processes, persistent within the examined age range (9–16 years). No significant effects were observed for the interaction of ADHD and the *SLC6A3* polymorphism, but the results revealed a significant main effect of *SLC6A3* genotype in the entire research sample. Subjects carrying 9R allele performed the switching task significantly worse in comparison to children with 10R/10R or 10R/11R genotype. *SLC6A3* polymorphism moderated age-related improvements in orienting and attentional switching. Results suggest that *SLC6A3* genotype influence these attentional/cognitive functions which deficits are not the key symptoms in ADHD.

## Introduction

Attention is usually conceptualized as a set of cognitive processes responsible for filtering and selecting behaviorally relevant information. Hence, it constitutes a precursor of other higher-level cognitive abilities such as learning and memory. In various cognitive models of attention, information selection operates in two modes: automatic or bottom-up versus controlled or top-down^1^. The bottom-up mode is driven by salient stimuli, which evoke automatic allocation of attention. In contrast, top-down processes rely on goals, cognitive strategies and effortful control, aiming to prioritize some stimuli over others. This distinction is clearly reflected in the Posner and Petersen (1990) model of attention, where three functionally and anatomically independent networks are proposed, each of them modulated by a different neurotransmitter^2^. According to this model, alerting is controlled mainly by acetylcholine, orienting by norepinephrine, and executive attention is dependent on dopamine (DA)^3^. It has been suggested that prefrontal DA and dopaminergic system-related genes play a dominant role in modulating top-down, but not bottom-up attention^4^.

The quality of attentional functioning varies widely amongst individuals and might also be significantly impaired in some psychiatric disorders, sometimes to the extent suggesting treating it as an endophenotype of the disease, like the Attention-Deficit/Hyperactivity Disorder (ADHD). Even though a variety of executive functions is impaired in children with ADHD, these deficits are not ADHD-specific^5^. They might also occur in children with other conditions and even in a healthy population. It has also been shown that children with ADHD may not exhibit impairments of executive functions^6^. These facts highlight the relevance of searching for the sources of variation in attentional functioning.

While experience during development, education and other environmental factors might explain some variation in cognitive functions, we know that it is partly determined by genetic factors^4,7,8^. Investigations of the genetic basis of neuropsychiatric disorders focused on variants in genes regulating neurotransmitter efficiency, particularly DA neurotransmission^9^. The most important DA regulator is the DA transporter (DAT)^10^ as it terminates DA signaling at the synapse through reuptake of DA into presynaptic terminals^11^. Thereby DAT regulates the concentrations of both extracellular DA at the synapse and intracellular DA within the presynaptic neuron, and modulates spatial and temporal dynamics of the DA signal^12–14^. Therefore, variations in the activity, density, levels of expression and function of DAT provide critical determinants of the synaptic concentration, the availability and also function of DA^15^. Through DA homeostasis, DAT preserves normal neurological function within the dopaminergic pathways of the central nervous system^16^. In prefrontal areas norepinephrine transporter (NET), is also capable of mediating the reuptake of DA, which indicates its importance in regulating prefrontal brain activity^17^. Both DAT and NET are established targets of therapeutics. Atomoxetine is a selective NET blocker, whereas psychostimulants exert their action via interference with both transporters function, resulting in an increase of extracellular DA and norepinephrine levels^18–21^.

A functional 40 bp variable number of tandem repeat (VNTR) polymorphism was identified in the human gene coding DAT (*SLC6A3* gene; also known as *DAT1*), located in the 3′ untranslated region (3′ UTR), with repeat numbers between 3 and 13. The 9- and 10-repeat (9R and 10R) alleles are the most frequent in the population^22^. Imaging studies in non-human primates have documented differences in DAT expression that can correlate with VNTR genotype^23^. In humans it has been linked to striatal DAT availability, however the findings are inconsistent. Meta-analyses suggest that the 9-repeat allele is associated with higher DAT activity in the striatal brain regions when compared against the 10-repeat allele^24,25^.

Studies on the genetic basis of differences in attention in healthy individuals suggested the link of *SLC6A3* polymorphism to executive attention^8,26^. Homozygosity for the 10R allele correlated with worse response inhibition^27^ and higher rates of impulsive errors in the Continuous Performance Task^28^. Carriers of the *SLC6A3* 10R allele performed better in procedures engaging top-down attention, and control of interference, but not in those assessing bottom-up attention^4,26,29^. However, a recent meta-analysis demonstrated no effects of *SLC6A3* on cognitive functions, including attention, in healthy adults^30^.

Many studies have investigated the association of this VNTR with ADHD, but with highly variable results. The 10R/10R genotype of *SLC6A3* is thought to be a risk factor for ADHD in children^31^, the 9R/9R genotype is associated with persistent ADHD in adulthood^32^. Barkley *et al*.^33^ reported 9R/10R genotype as reliably associated with symptoms of ADHD (hyperactivity, impulsivity, externalizing and pervasive behavioral problems in both children and adolescents)^33^. A recent study questioned the association of *SLC6A3* gene polymorphism with ADHD while indicating at the same time that the 10R/10R genotype in patients with ADHD affects processing speed and cognitive flexibility^34^. Similarly, an earlier study on ADHD reported that children homozygous for the *SLC6A3* 10R allele performed more poorly on a sustained attention task than subjects with other genotypes^35^.

The effect of age has been rarely considered when studying the relationship between ADHD phenotype, neurocognitive functioning and the genetic factors. Expression levels of genes can differ across different stages of development^36^, with the decline of the DAT availability with age reported most frequently^37^. Worth noticing here, longitudinal studies on ADHD patients have shown that symptoms of hyperactivity significantly decrease with age^38^. As the contribution of risk genes to ADHD may not be constant across the life course, the age should be considered an important factor in the analysis of genetic underpinnings of ADHD.

The aim of the study was twofold. First, we focused on the relationship between *SLC6A3* and individual differences in attentional processes of healthy and ADHD children and teenagers. We assessed the broad spectrum of these processes with a battery of attentional tests and computerized procedures including measures of sustained and selective attention, attentional switching and efficiency of three attentional networks responsible for alerting, orienting and executive attention. Second, we investigated the moderating role of age and *SLC6A3* 3′ UTR VNTR variants in diagnosis-related effects.

## Materials and Methods

### Subjects

A total of 150 children and teenagers (Caucasian), aged 9–16 participated in the study. The clinical group consisted of 74 participants with an ADHD diagnosis (M_age_= 13.11 ± 2.04; 12 females). The healthy control group was age- and sex-matched to ADHD group (n = 76; M_age_ = 13.15 ± 2.23; 12 females). ADHD subjects were recruited among outpatients of the psychiatry clinic at Public Pediatric Teaching Hospital in Warsaw, Poland. The diagnosis was conducted at the clinic by an experienced team of psychiatrists and psychologists according to the diagnostic criteria of the DSM-IV TR (4th edition, text revision; American Psychiatric Association, 2000) as previously described^39^ and included: an interview with patients’ parents, Diagnostic Structured Interview for ADHD and Hyperkinetic Disorder according to ICD-10 and DSM-IV TR^40^, the Behavioral Disorders Supplement of Diagnostic Interview Kiddie-SADS-Present and Lifetime Version, as well as observation of patients’ behavior. The intensity of the ADHD symptoms was rated by both parents and teachers using the ADHD Rating Scale^40^. The comorbidity diagnosis was based on the diagnostic criteria for ICD-10 (World Health Organization, 1994), and the diagnosis was verified during no less than three appointments. If the results of all diagnostic methods were consistent, the diagnosis was confirmed, and such patients were invited to take part in our study. The ADHD group included children diagnosed with combined (n = 53) or predominantly inattentive ADHD subtypes (n = 21). In 66% of ADHD participants at least one comorbidity was diagnosed. The most common was oppositional defiant disorder (ODD) present in 47% of ADHD participants. 28% of patients were diagnosed with specific developmental disorders of scholastic skills. The inclusion criteria for our ADHD group were: a confirmed diagnosis, no previous head injuries with a loss of consciousness, and no neurological disease (e.g. epilepsy) or psychiatric disorders, except for ODD or specific school disabilities (e.g. dyslexia), which are highly comorbid in ADHD cohorts. All ADHD participants were asked to abstain from taking stimulant medication at least 24 h before testing.

A healthy control group was recruited from among Warsaw school’s students. Parents completed a questionnaire providing child’s health condition information, which was used to select participants who did not report any attentional problems, psychiatric or neurological diagnosis, brain injuries with loss of consciousness, somatic disorders, and had no close family members with ADHD diagnosis.

The study was approved by the local Ethics Committee at the SWPS University of Social Sciences and Humanities and the Medical University of Warsaw. All participants provided assent, and parents gave informed written consent in accordance with the Declaration of Helsinki.

### Isolation of DNA and genotyping

Subjects provided a saliva sample into an Oragene collection and preservation kit (DNA Genotek, Inc., Kanata, Ontario, Canada). Genomic DNA was isolated using extraction kit (Swab, A&A Biotechnology, Poland) according to the manufacturer’s instructions. The *SLC6A3* polymorphism analysis was performed by polymerase chain reaction (C1000 Touch Thermal Cycler, BioRad, USA) with a final reaction volume of 20 µL [approximately 30 ng of DNA, One*Taq*® Hot Start Quick-Load® 2X Master Mix with Standard Buffer (#M0488S, NEB)] and 0.2 µM of each primer: F 5′-TGTGGTGTAGGGAACGGCCTGAG‐3′ and R 5′-CTTCCTGGAGGTCACGGCTCAAGG‐3′^41^. Thermal cycling consisted of a 2 min initial denaturation phase at 94 °C followed by 35 cycles of 30 s at 94 °C, 60 s at the annealing temperature of 60 °C, 45 s at 68 °C, and a final extension step of 5 min at 68 °C. Amplified DNA was separated on a 2% agarose gel and visualized using the Gel Doc EZ system (Bio-Rad, USA). Five alleles of the *SLC6A3* were observed: the 7-repeat (360 bp), 8-repeat (400 bp), 9-repeat (440 bp), 10-repeat (480 bp) and 11-repeat (520 bp). Genotypes for all participants were collapsed into categories based on the presence or absence of the 9R allele (Table 1). The distribution of genotypes (including two main alleles 9R and 10R) calculated for Control group was in Hardy-Weinberg equilibrium (χ^2^ = 1.99, p = 0.157).

**Table 1:** Genotype distribution of 40-bp VNTR polymorphism in ADHD and controls.

	SLC6A3 gene					
	9R allele		Genotype			
	+	−	9R/9R	9R/10R	10R/10R	other
n_ADHD_	37	37	2	34	36	2
n_Control_	38	38	4	34	36	2
n_Total_	75	75	6	68	72	4

Other: ADHD: 7R/9R and 10R/11R; Control: 8R/9R and 7R/9R.

For further analyses subjects were divided into two Genotype groups: the 9R group included 9R/9R (n = 6), 9R/10R (n = 68), 8R/9R (n = 1) and 7R/9R (n = 2) genotypes; the 10R group included 10R/10R (n = 72) and 10R/11R (n = 1) genotypes.

### Cognitive measures

The cognitive diagnosis included two computerized procedures and one clinical battery of tests. The choice of measures allowed us to cover all essential facets of attentional functioning using well-validated measures.

#### Sustained Attention to Response Task (SART)

**Figure 1:**
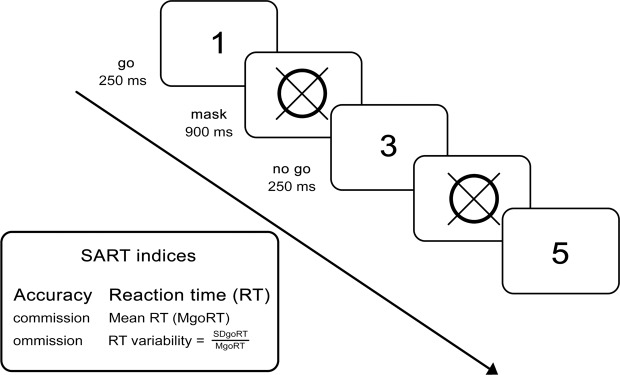
Sustained Attention to Response Task (SART). Participants’ task is to respond to every digit except digit “3”, which is presented rarely.

To measure sustained attention, we used SART^42^. In this task, randomly selected single-digit numbers are displayed on a computer screen. The subject is asked to respond as fast as possible to every digit (go trial) except the digit ‘3’ (no-go trial, target). Responses are given by pressing a computer mouse button with the index finger of the dominant hand. When digit ‘3’ is presented, the subject is asked to withhold the motor reaction. A total number of 200 go, and 25 target trials were presented preceded by 25 training trials. The procedure is presented in Fig. 1.

Attention effectiveness measures included accuracy and reaction time (RT) indices such as: commission (failure to withhold response to the target) and omission (failure to respond to go digits) errors, mean RT for correct go-trial responses (GoRT), and RT variability (defined as the SDgoRT/MgoRT).

#### Attention Network Test (ANT)

**Figure 2:**
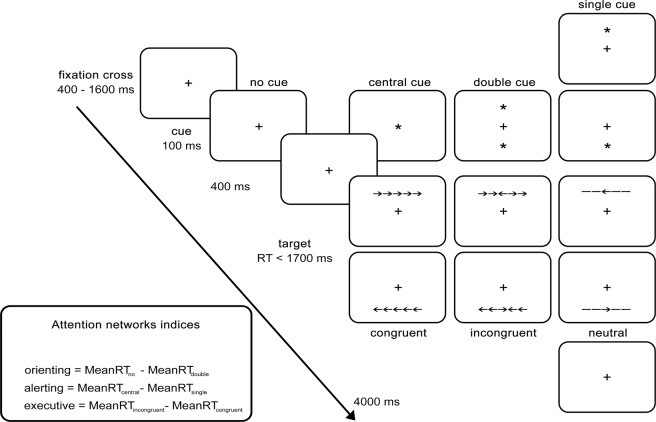
Attention Network Test (ANT). Participants’ task is to discriminate the direction of the central arrow (target). Before the target presentation, none or one variant of a cue is provided, either temporally informative or temporally and spatially informative. Target is presented in one of three variants - congruent, incongruent or neutral.

The ANT combines cued RT^43^ and flanker tasks^44^. We used a classic variant of the task as proposed by Fan *et al*.^45^ (Fig. 2). Participants’ task was to indicate the direction of the central arrow (target) regardless of flanking stimuli (either congruent, incongruent or neutral), by pressing a left or right computer mouse button. Before each target presentation, a cue was provided (4 variants, either temporally or temporally and spatially informative, Fig. 2). Both accuracy and RTs were recorded. The procedure consisted of a training block (24 trials) and the main part including 3 blocks of 96 trial each. The order of trial types was randomized. Effectivity of attentional networks was determined according to standard formulas (Fig. 2)^45^.

#### Test of Everyday Attention for Children (TEA-Ch)

The TEA-Ch was designed to assess various components of attention in children^46^. The TEA-Ch comprises of nine subtests that are used to measure focused (selective) attention (*Sky Search -* SSG and *Map Mission -* MapMu), sustained attention (*Walk don’t Walk* - WdW, *Score*!- Score, *Sky Search Dual Task*, *Score!DT*, *Code Transmission*), and attentional control/switching (*Creature Counting -* CL and *Opposite Worlds -* OA). We did not use the *Code Transmission and Score DT*, which involve the processing of verbal material and Polish language versions are unavailable. The scores obtained in the *Sky Search Dual Task* were dropped from the analysis, due to serious problems with following the test instructions by the majority of participants.

### Statistics

A series of linear regression models were estimated with genotype variant (9 vs 10), diagnosis (ADHD vs control), age (centered), and all their interactions as predictors of cognitive performance. A separate model was estimated for each of the attentional indices described above. In the encoding of nominal variables, sum contrasts were used to facilitate the interpretation of lower-order effects. Due to the presence of heteroscedasticity, all p-values were estimated using heteroscedasticity-consistent standard errors^47^. R environment ver. 3.4.2^48^ was used in all analyses.

Our design offered satisfactory power (80%) in detecting effects larger than f^2^ = 0.06 (alpha = 0.05). Power calculations were performed in G*POWER (ver. 3.1)^49^. Calculations were based on the assumption that one extra effect is added to a model containing 5 predictors and R^2^ for the full model equals 0.20. (In the power analysis we decided to use conservative estimates of R^2^ values based on simpler models not including the effects of genotype based on our own data, as no comparable literature was available).

### Data filtering

Due to a large number of dependent variables, outlier removal criteria were applied on

a task-by-task basis leading to small fluctuations in the final sample size across analyses.

In the case of ANT prior to analysis for every subject we removed RTs for correct trials exceeding three standard deviations (SD) above or below the mean. Afterwards, we excluded from the analysis subjects whose performance indices exceeded 3 SD above or below the mean of overall accuracy in the task or mean networks score (orienting, altering, executive).

In SART before RTs calculation we removed RTs faster than 100 ms. Then outliers were defined based on their mean accuracy and RT indices - if the mean values were higher or lower than 3 SD. In the TEA-Ch no filtering criteria were applied, however small differences in sample size are related to the missing scores in some of the subtests (participants were not following the instructions, some of the indices might be computed only if participants reach certain performance criterion etc.). The number of participants included in the analysis for each dependent variable is reported in Tables 2 and 3.

**Table 2:** Linear regression analysis for all attentional indices predicted by age, diagnosis, and genotype group including all main effects and two-way interactions.

effect	age		diagnosis		genotype		age × diagnosis		age × genotype		genotype × diagnosis		model		
task	Beta	t	Beta	t	Beta	t	Beta	t	Beta	t	Beta	t	adjR^2^	F	n
**TEA-Ch**															
SSG	**−0**.**30**	**−6**.06^***^	**−0**.**23**	**−2**.04*	−0.04	−0.34	0.01	0.23	0.21	0.43	−0.05	−0.45	**0**.**18**	**8**.53***	**148**
MapMu	**3**.**13**	**8**.29***	**2**.**75**	**3**.23**	0.004	0.005	0.72	1.88	0.53	1.44	0.50	0.58	**0**.**34**	**20**.2***	**148**
Score	**0**.**21**	**3**.32**	**0**.**27**	**2**.04*	−0.10	−0.76	−0.03	−0.48	−0.04	−0.72	−0.09	−0.48	**0**.**08**	**3**.89**	**147**
WdW	**0**.**44**	**3**.69***	**1**.**57**	**6**.08***	−0.27	−1.04	−0.07	−0.66	−0.64	−0.59	0.15	0.57	**0**.**25**	**11**.33***	**146**
CL	**−0**.**25**	**−4**.97***	**−0**.**45**	**−4**.99***	**0**.**18**	**2**.14**	0.04	0.75	−0.003	−0.07	0.09	1.08	**0**.**33**	**14**.1***	**139**
OA	**−1**.**29**	**−5**.85***	**−1**.**02**	**2**.70**	**0**.**92**	**2**.47*	−0.18	−0.85	**−0**.**52**	**−2**.59*	0.07	0.20	**0**.**35**	**13**.19***	**146**
**ANT**															
Total ACC	**0**.**004**	**2**.71**	**0**.**008**	**2**.79**	0.005	1.53	−0.002	−1.02	−0.002	−1.74	−0.002	−0.69	**0**.**104**	**2**.48*	**142**
orienting	−1.92	−0.97	1.89	0.57	**9**.**57**	**2**.88**	−2.27	−1.14	**−5**.**21**	**2**.85**	−1.31	−0.39	**0**.**12**	**2**.87*	**142**
alerting	**−4**.**37**	**−2**.37*	**−7**.**08**	**−2**.16*	−4.36	−1.31	1.12	0.61	1.50	0.90	0.20	0.06	**0**.**07**	**2**.27*	**142**
executive	**−7**.**57**	**−2**.57*	**−15**.**92**	**−2**.53*	−8.16	−1.26	−0.14	0.05	3.63	1.34	1.12	0.17	**0**.**07**	**3**.84**	**142**
**SART**															
MRT	−7.10	−1.57	7.66	0.87	−0.48	−0.05	4.72	1.05	1.02	0.23	−2.80	−0.32	<0	<1	145
Coeff RT	**−0**.**02**	**−2**.53*	**−0**.**08**	**−4**.91***	0.01	0.66	−0.01	−1.66	−0.01	−1.13	0.02	1.35	**0**.**202**	**10**.76***	**145**
ommision	**−0**.**01**	**−3**.05**	**−0**.**03**	**−5**.13***	0.004	0.64	−0.003	−1.37	−0.004	−1.71	0.001	0.10	**0**.**2**	**9**.18***	**145**
commision	**−0**.**03**	**−3**.51***	**−0**.**08**	**−4**.11***	0.01	0.72	−0.01	−1.26	−0.001	−0.97	0.002	0.11	**0**.**17**	**6**.07***	**145**

Description.

***p < 0.001; **p < 0.01; *p < 0.05; significant effects and models indicated in bold.

TEA-Ch – Test of Everyday Attention for Children; ANT – Attention Network Test; SART – Sustained Attention to Response Task; SSG - Sky Search; MapMu - Map Mission; CL - Creature Counting; OA - Opposite Worlds; WdW – Walk don’t Walk subtest; Total ACC – Overall accuracy in ANT task; MRT – Mean Reaction Time, Coeff RT – Coefficient of Reaction Time Variation.

**Table 3:** Descriptive statistics for scores in TEA-Ch, ANT and SART.

task performance index		ADHD			Control			All		
		n	M	SD	n	M	SD	n	M	SD
TEA-Ch	SSG	73	3.67	1.54	76	3.21	1.31	148	3.43	1.44
	MapMu	73	38.82	11.96	76	44.20	12.35	148	41.56	12.42
	Score	72	8.36	1.72	76	8.87	1.40	147	8.62	1.58
	WdW	72	12.75	3.63	75	15.92	2.64	146	14.37	3.53
	CL	68	3.94	1.32	71	3.10	0.97	139	3.51	1.22
	OA	71	27.33	5.64	76	25.48	5.11	146	26.37	5.43
ANT	Total ACC	67	0.96	0.04	75	0.97	0.02	142	0.97	0.04
	orienting		47.47	45.15		52.22	36.44		49.98	40.70
	alerting		47.50	46.97		32.71	26.79		39.69	38.26
	executive		151.58	79.63		118.48	65.94		134.1	74.33
SART	M RT	71	362.26	104.64	74	377.54	99.97	145	370.06	102.21
	Coeff RT		0.58	0.23		0.41	0.19		0.50	0.23
	ommision		0.10	0.08		0.04	0.05		0.07	0.07
	commision		0.59	0.22		0.44	0.24		0.51	0.25

Description.

TEA-Ch – Test of Everyday Attention for Children; ANT – Attention Network Test; SART – Sustained Attention to Response Task; SSG - Sky Search; MapMu - Map Mission; CL - Creature Counting; OA - Opposite Worlds; WdW – Walk don’t Walk subtest; Total ACC – Overall accuracy in ANT task; MRT – Mean Reaction Time, Coeff RT – Coefficient of Reaction Time Variation.

## Results

As none of the regression models revealed a significant third level interaction effects of Age, ADHD diagnosis and Genotype (all *p*-values for the interaction > 0.1), we decided to report detailed results of more parsimonious models including main effects, and all two-way interactions. Detailed results are reported in Table 2. Descriptive statistics for all dependent variables are presented in Table 3.

### Genotype interacts with age

**Figure 3:**
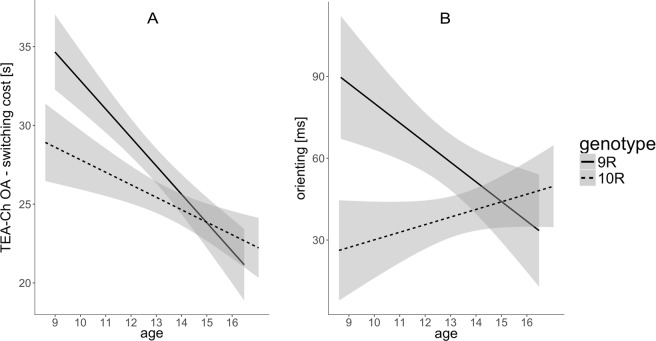
Second level interaction effect of Genotype and Age observed for (**A**) switching assessed with OA subtest of TEA-Ch and for (**B**) orienting measured with ANT. *SLC6A3* polymorphism moderates development of attention processes.

The analysis revealed a significant interaction of Age and Genotype for OA subtest measuring switching costs. The analysis showed age-related improvement for both genotype variants. Nevertheless, subjects with 9R allele started from a significantly worse level than 10R allele group subjects, and reached the 10R group performance level at around 15 y.o. age (Fig. 3A).

A similar effect was observed for orienting index measured with ANT. Subjects with 9R allele displayed less efficient orienting at a younger age, but with development improved and reached a similar level of performance as subjects from group 10R (Fig. 3B). Both interaction effects involving Genotype and Age effects are presented in Table 2 and Fig. 3.

**Figure 4:**
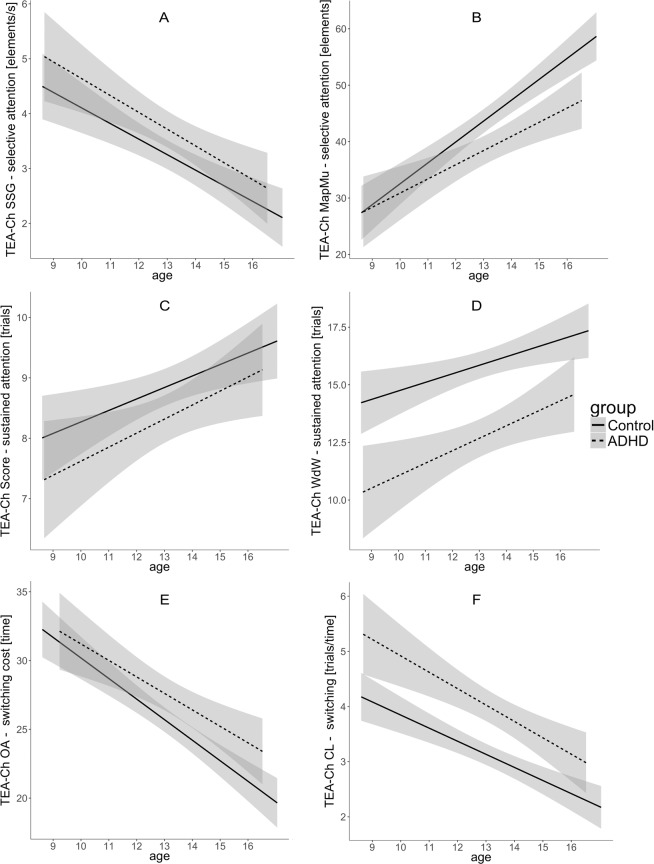
Main effects of Age and Diagnosis for selective attention (**A**,**B**), sustained attention (**C**,**D**) and switching (**E**,**F**) measured with subtests of TEA-Ch. Significant effects of attention processes development observed in both groups; ADHD’s group performance less efficient in comparison to Controls. SSG - Sky Search; MapMu - Map Mission; WdW - Walk don’t Walk; OA - Opposite Worlds; CL - Creature Counting.

We also observed a significant main effect of genotype for CL – switching task showing that mean-aged subjects carrying 9R allele were characterized by less efficient switching abilities in comparison to 10R group participants (Table 2, Fig. 4F).

### Main effects of ADHD diagnosis and Age

**Figure 5:**
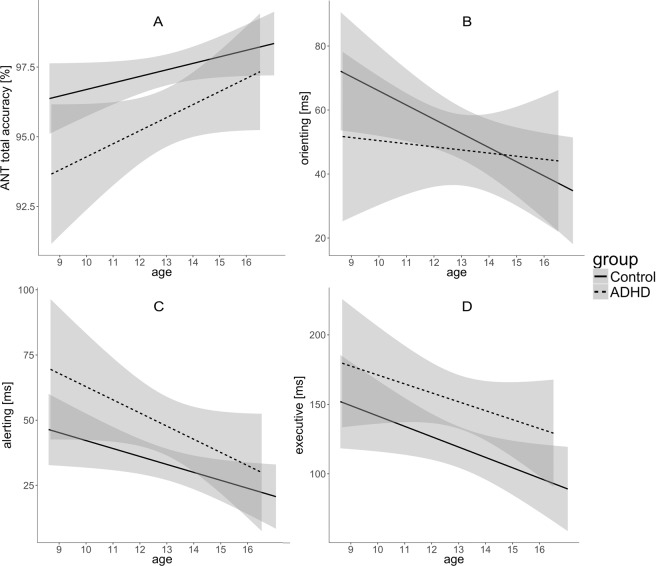
Main effects of Age and Diagnosis observed for overall accuracy and attention networks measured with ANT. (**B**) Orienting network efficiency does not improve with age, and does not differentiate compared groups. Effect of processes development observed for (**A**) overall accuracy and for (**C**) alerting and (**D**) executive networks in both groups. (**B**) Orienting network efficiency does not improve with age, and does not differentiate compared groups. Lower accuracy and less efficient alerting and executive networks in AHD group in comparison to Controls.

**Figure 6:**
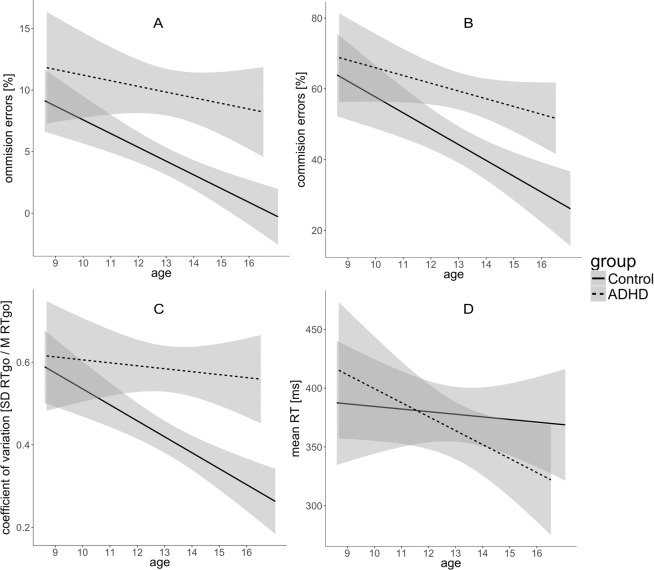
Main effects of Age and Diagnosis observed for indices of sustained attention measured with SART. Accuracy (**A**,**B**) and RT variability (**C**) improves with age in both groups, but ADHD group constantly perform less accurate and more variably in comparison to Controls. (**D**) No development nor group difference for simple, mean RT observed.

The analysis did not reveal any significant Age x ADHD diagnosis effects. Nonetheless, a significant main effect of age indicating developmental improvement was present in almost all indices of attentional processes. Linear improvement with age was found for: all subtest of TEA-Ch (Table 2, Fig. 4); alerting and executive attention indices and total accuracy in ANT (Table 2, Fig. 5A,C,D); accuracy and RT variability indices measured with SART (Table 2, Fig. 6A–C).

Furthermore, we observed that the ADHD group performed significantly worse in comparison to Controls in most of the tasks. The ADHD showed deficits in switching (CL, OA), sustained attention (WdW) and selective attention (MapMu) measured by TEA-Ch (Table 2, Fig. 4).

The main effect of group was also significant for total accuracy in ANT, and effectiveness of executive attention and alerting indicating deficits of these processes in ADHD group in comparison to Controls but not in orienting processes (for details see Table 2 and Fig. 5).

The analysis revealed the main effect of ADHD diagnosis on the number of commission and omission errors in SART as well as coefficient of RT variation (Table 2, Fig. 6A–C). The ADHD patients displayed less accurate and more “variable” performance in comparison to healthy controls.

### No interaction of ADHD diagnosis and Genotype

The analysis did not reveal any significant Genotype x ADHD diagnosis interactions (Table 2.)

## Discussion

The main goal of the study was to determine the joint contribution of *SLC6A3* 3′ UTR 40-bp VNTR variants and age in explaining individual differences in attentional functioning of healthy and ADHD-affected children and teenagers. To the best of our knowledge, this is the first study on ADHD and control cohort using such a detailed and wide assessment of the attention and regression approach to analyze in more details how the attention functioning changes with age and what is the contribution of *SLC6A3* 3′ UTR VNTR variants in these processes. The *SLC6A3* gene polymorphism was not associated with the presence of ADHD, and its distribution was similar in compared groups. Furthermore, *SLC6A3* 3′ UTR VNTR variants effects did not interact with diagnosis in explaining the quality of attentional functioning. However, its effects were observed for switching and orienting efficiency, showing that 9R carriers displayed worse performance in comparison to children with 10R/10R or 10R/11R genotype. At the same time, a typical developmental improvement in attentional processes was observed in almost all measured indices. *SLC6A3* gene polymorphism moderated developmental improvements in orienting and attentional switching. The effects of age did not interact with ADHD diagnosis - the ADHD group performed consistently worse in comparison to controls in most of the tasks, demonstrating pronounced deficits across various domains of attention.

### Genotype effect

Significant effect of the *SLC6A3* genotype showed that subjects with the 9R allele, when compared to those from the 10R group, were characterized by less efficient orienting processes measured with the ANT task and obtained significantly worse results in the TEA-Ch subtests evaluating attentional switching (CL, OA). The association between *SLC6A3* 10R/10R or 10R/11R genotypes and better performance in cognitive tasks^50^ appears counterintuitive, as the 10R allele has been reported as the risk allele for ADHD^51^, and related to poorer performance in cognitive tasks^27,28^. However, the debate on the role of 10R is far from being settled with studies reporting both opposite^29,51^ and null^30^ effects. In our study, we did not observe effects of genotype on sustained attention neither in the SART nor for the executive attention assessed with the ANT. However, *SLC6A3* genotype showed significant associations with the specific orienting measure in the ANT. These results are in contrast to the reports showing associations of *SLC6A3* with executive aspects of attention, and not with orienting^7,26^ or those suggesting no association between dopaminergic genes and orienting^52^. However, more recent data provide evidence linking DA signalling^53^ and the *SLC6A3* gene^54^ with involuntary orienting.

It is important to underline, that intentionally controlled attention shifting/switching shares some of the neural underpinnings with orienting. Firstly, similarities among neuroimaging brain activations for shifting and orienting have been reported^55^. Secondly, there is evidence that set-shifting and other executive functions, such as cognitive flexibility and attention, are mediated by dopaminergic activity^56^, and that DAT plays an important role in these functions, as was shown in the animal model of ADHD^57,58^. In contrast to the study by Garcia-Garcia *et al*. (2010), who reported that individuals genotyped 10R/10R show more rigid behavior in a task-switching protocol when compared to participants with the 9R allele, we have shown that 10R/10R and 10R/11R children outperform children with 9R allele on TEA-Ch subtests assessing switching abilities (CL and OA)^59^. It has been shown previously that lower levels of DAT availability are associated with neurocognitive deficits^60^. The 10R allele, compared to the 9R allele, has been associated with increased transporter protein expression both *in vitro*^61^, and in imaging research in humans *in vivo*^25^; also the DAT mRNA expression in postmortem midbrain tissue was shown higher for homozygous 10R carriers^62^. However, results showing the opposite allelic associations have been reported^63^ and meta-analysis of neuroimaging studies shown increased DAT activity for 9R carriers in striatal brain regions^24,25^. Genotype variant moderated the effect of age in our study, such that 9R allele carriers started from a significantly worse level than the 10R group, but finally reached the same level at a higher age.

### Age effect

Almost all indices of attentional processes revealed a typical age-related improvements; the only exceptions were orienting and average RT measures in SART. The orienting network starts to develop very early in newborns^64^ and no significant age changes in children 7–11 y.o. have been reported^65^. Our results revealed changes with age in orienting moderated by *SLC6A3* genotype. At the youngest age, *SLC6A3* 9R allele carriers performed worse in comparison to 10R group subjects but improved their performance with increasing age; whereas the performance of 10R group did not significantly change with age. Both alerting and executive attention assessed with ANT improved with age. The alerting network starts developing early, but the network undergoes further development during late childhood in relation to maturation of frontal cortex^66^. The executive attention network develops later, during childhood and in early adolescence^67^, which is related to the progressive maturation of the anterior cingulate cortex and lateral prefrontal cortex^66^.

The effect of age was significant for all TEA-Ch subtests. Improvements in (non normalized) results with increasing age were expected, as the TEA-Ch has been found to be an age-sensitive tool^68^. These changes reflect higher effectiveness of voluntary attentional control in older subjects, as it is well established that throughout development endogenous factors come to play an increasing role in determining the locus of attention^67^.

Research on age-related changes in performance on the SART task is scarce. Measures of sustained attention improve rapidly during the early school-age years and modestly after the age of 12^69^. Our results, showing age effect in the omission and commission errors number and RT variability, are in agreement with these reports. The lack of effect of age on mean RT might be related to the speed-accuracy tradeoff and certain ambiguity of this particular performance index in SART. Faster average performance in SART might reflect more effective control of the motor reactions, but also poorer inhibitory control. These two factors cannot be easily disentangled and - therefore - mean RT is usually not treated as key performance measure in SART.

### Diagnosis effect

The results of ANT showing poorer alerting and executive attention in ADHD children in comparison with controls and no differences in orienting are in agreement with previous reports^70^. They are also congruent with Posner and Raichle’s^71^ statement accentuating lack of empirical support for the involvement of the orienting network in the psychopathology of ADHD.

The poorer performance of ADHD children in comparison with controls in SART, as observed in our study, has been reported before^72^ and is fully consistent with other research on sustained attention in children with ADHD^73^. High within-subject intertrial variability, fluctuations in attention, is the most marked symptom of ADHD^74^. Contrarily to the Bellgrove *et al*. ^35^, we did not observe significant genotype-diagnosis interaction. However, it is important to note that our study involved much larger sample and controlled the effects of *SLC6A3* in both studied groups (not just in the ADHD subjects) which was not the case in the cited study.

Differences between ADHD patients and controls on certain subtests of TEA-Ch were found before. However, the pattern of attention deficits in ADHD across studies was inconsistent^e.g.^ ^68,75,76^. ADHD is neuropsychologically and cognitively heterogeneous condition^77^, however, impairments in specific attentional abilities, such as sustained attention and executive attentional functions, seem to be particularly pronounced^5^. The results of our study, suggesting the presence of deficits in almost all attentional functions measured with three tests used in the study, are in line with hypotheses suggesting the existence of multiple deficits in ADHD^78^. Our analyses did not reveal significant interactions of Age and ADHD Diagnosis for any measure of attention. Instead, we observed two additive effects of Age and Diagnosis. The developmental pattern of attentional functions was similar in ADHD and healthy controls, and the lack of interaction implies the presence of persistent attentional deficits in ADHD subjects within the examined age range.

The lack of interaction of Genotype and Diagnosis and the effect of Genotype but not Diagnosis for orienting suggest that the *SLC6A3* gene polymorphism influence these attentional/cognitive functions which are not involved in the emergence of ADHD symptomatology. For switching, both Genotype and Diagnosis effects have been revealed. However, explanations for poor performance of ADHD subjects in CL and OA tasks might include motivational factors leading to delay aversion^79^ and non-optimal regulation of activation during task performance^80^ - thus deficits in functions distinct from the ones influenced by *SLC6A3* gene polymorphism.

### Limitations

Some limitations of our study should be mentioned. Firstly, our sample size might offer statistical power too low to investigate subtle genetic influences. Distribution of sex and ADHD subtypes did not allow us to analyse their effects. However, we run supplementary analysis on male participants only and its results closely resembled the ones reported in the paper. In particular, there were no changes in any of the effects involving the genotype. Additional analysis within the ADHD group did not reveal any effect involving subtypes, which might be explained by either the limited sample size and power of between-subtype comparisons, or non-existence of such effect^81^. Secondly, the cross-sectional character of the design did not allow us to study the dynamics of developmental changes in attentional functioning. Future longitudinal research investigating the relationship between genotype, changes in ADHD symptomatology and the development of attentional processes would be necessary to establish more clearly the causal contribution of all these factors.

Nonetheless, we still find regression-based approach more appropriate as the possibility of studying all the higher-level interactions allows us to address, at least partially, the limitations of simple between-group comparisons used in the majority of existing literature. It is also important to justify our decision not to report familywise error-corrected p-values, even though we ran multiple regression models. It was motivated by the scarcity of data on the interplay of *SLC6A3* polymorphism, age and ADHD diagnosis as well as the exploratory nature of our study. We are aware that some of the reported results might be false-positives. However, this approach increased the chances of revealing significant effects of *SLC6A3*. In this context, the fact that not a single interaction involving both *SLC6A3* and ADHD was detected, speaks strongly for the main thesis of our paper, that is the independence of the effects of dopamine transporter gene and ADHD diagnosis in shaping attentional functioning across the studied age-range.

Patients were not drug-naive, hence they were asked to abstain from taking medication at least 24 h before testing, since methylphenidate (MPH) plasma concentration 24 h after drug administration is close to zero^82,83^. It has been shown previously that ADHD children following MPH withdrawal display deficits in comparison with both healthy controls and with ADHD children on MPH^84^. One could argue that the withdrawal might cause attentional impairments as a pharmacological effect. To our knowledge, there are no studies examining differences between the cognitive performance of children with ADHD prior to the commencement of MPH treatment and following a discontinuation of the medication. Nonetheless it has been shown that MPH treatment improves performance of ADHD children, when compared with healthy controls, however, children with ADHD does not reach an undisturbed level of attention^80^. The main goal of our study was to establish the role of SLC6A3 gene polymorphism in attentional functioning. Meta-analysis^85^ showed that there is no significant association between SLC6A3 polymorphism and response to MPH treatment. The meta-analysis also found no effects on dimensions of hyperactivity/impulsivity and inattention. Therefore we can assume that the medication effects could not influence the main results of the study.

## Conclusions

This study is a significant contribution to the literature on the relationship between attentional functioning, its deficits in ADHD and the SLC6A3 3′ UTR 40-bp VNTR variants by examining additionally the effect of age. The developmental pattern of attentional functions was similar in ADHD and healthy controls. However, deficits observed in ADHD subjects were persistent within the examined age range. No significant effects were observed for the interaction of ADHD and the SLC6A3 polymorphism. The *SLC6A3* polymorphism moderated age-related improvements in orienting and attentional switching in the whole research sample, independently from ADHD diagnosis.

## Supplementary information

Supplementary table.

## Data Availability

The datasets generated during and/or analysed during the current study are available from the corresponding author on reasonable request.
