# Supplementary material for: The SLC6A3 gene polymorphism is related to the development of attentional functions but not to ADHD
Source: Sci Rep. 2020 Apr 10;10:6176. doi: 10.1038/s41598-020-63296-x (PMC7148317; doi:10.1038/s41598-020-63296-x)
Supplement: Supplementary file 1 — Supplementary table. [file 41598_2020_63296_MOESM1_ESM.pdf]

The *SLC6A3* gene polymorphism is related to the development of attentional functions but not to ADHD

Katarzyna Kuc[1], Maksymilian Bielecki[1], Ewa Racicka-Pawlukiewicz[2], Michał B. Czerwinski[3], Anita Cybulska-Klosowicz[4]

1. Department of Psychology, SWPS University of Social Sciences and Humanities, Warsaw, Poland

2. Department of Child Psychiatry, Medical University of Warsaw, Warsaw, Poland

3. Laboratory of Neuroinformatics, Nencki Institute of Experimental Biology, Polish Academy of Sciences, Warsaw, Poland

4. Laboratory of Neuroplasticity, Nencki Institute of Experimental Biology, Polish Academy of Sciences, Warsaw, Poland

Linear regression analysis for all attentional indices predicted by age, diagnosis, and genotype group including all main effects and two-way interactions **performed for male participants only.**

**effect**	age		diagnosis		genotype		age x diagnosis		age x genotype		genotype x diagnosis		model		
**task**	Beta	t	Beta	t	Beta	t	Beta	t	Beta	t	Beta	t	adjR^2	F	n
**TEA-Ch**															
SSG	**-0.26**	**-5.50*****	-0.06	-1.36	-0.06	-0.52	-0.02	-0.31	0.001	0.01	-0.14	-1.15	**0.19**	**6.93*****	**123**
MapMu	**3.18**	**7.89*****	**2.92**	**3.25****	-0.38	-0.42	0.70	1.62	0.27	0.66	1.51	1.67	**0.41**	**16.13*****	**123**
Score	**0.22**	**3.33****	0.22	1.53	-0.02	-0.12	-0.08	-1.22	-0.08	-1.29	-0.12	-0.77	**0.13**	**3.06****	**123**
WdW	**0.41**	**3.10****	**1.60**	**5.63*****	-0.31	-1.05	-0.07	-0.57	-0.02	-0.14	0.23	0.79	**0.29**	**8.80*****	**122**
CL	**-0.25**	**-4.60*****	**-0.42**	**-4.16*****	**0.20**	**2.08***	0.04	0.80	-0.01	-0.22	0.08	0.84	**0.35**	**11.43*****	**118**
OA	**-1.31**	**-5.22*****	**-1.10**	**-2.58***	**0.93**	**2.18***	-0.19	-0.80	**-0.48**	**2.13***	-0.13	0.75	**0.38**	**11.00*****	**122**
**ANT**															
Total ACC	**0.003**	**2.13****	0.006	0.95	0.003	0.95	-0.0001	-0.71	-0.002	-1.28	-0.001	-0.31	**0.08**	**1.57***	**119**
orienting	-2.73	-1.27	0.19	0.05	**9.80**	**2.59***	-2.40	-1.13	**-5.89**	**-2.96****	-1.48	-0.39	**0.18**	**2.84****	**119**
alerting	**-5.01**	**-2.70****	-5.65	-1.50	0.31	0.08	1.08	0.56	2.56	1.37	-2.37	-0.58	**0.11**	**2.28***	**119**
executive	**-7.63**	**-2.26***	-11.97	-1.63	-8.45	-1.10	-0.01	0.004	3.61	1.13	0.06	0.01	**0.08**	**2.31****	**119**
**SART**															
MRT	-6.78	-1.31	10.83	1.08	-2.09	-0.20	5.27	1.03	1.69	0.34	-3.49	-0.34	< 0	< 1	120
Coeff RT	**-0.02**	**-1.94***	**-0.095**	**-4.97*****	0.003	0.167	-0.016	-1.62	-0.01	-1.05	0.02	1.15	**0.26**	**9.01*****	**120**
ommision	**-0.01**	**-2.60***	**-0.03**	**-4.90*****	-0.002	-0.32	-0.003	-1.14	-0.004	-1.44	0.0003	0.07	**0.24**	**7.52*****	**120**
commision	**-0.03**	**-2.76*****	**-0.08**	**-3.87*****	0.03	1.39	-0.014	-1.35	-0.009	-0.83	-0.008	-0.39	**0.22**	**5.40*****	**120**

Description

*** $p < 0.001$; ** $p < 0.01$; * $p < 0.05$; significant effects and models indicated in bold

TEA-Ch – Test of Everyday Attention for Children; ANT – Attention Network Test; SART – Sustained Attention to Response Task; SSG - Sky Search; MapMu - Map Mission; CL - Creature Counting; OA - Opposite Worlds; WdW – Walk don't Walk subtest; Total ACC – Overall accuracy in ANT task; MRT – Mean Reaction Time, Coeff RT – Coefficient of Reaction Time Variation
